# Treatment Outcome of Severe Acute Malnutrition Cases at the Tamale Teaching Hospital

**DOI:** 10.1155/2015/641784

**Published:** 2015-05-03

**Authors:** Mahama Saaka, Shaibu Mohammed Osman, Anthony Amponsem, Juventus B. Ziem, Alhassan Abdul-Mumin, Prosper Akanbong, Ernestina Yirkyio, Eliasu Yakubu, Sean Ervin

**Affiliations:** ^1^School of Medicine and Health Sciences, University for Development Studies, P.O. Box 1883, Tamale, Ghana; ^2^Tamale Teaching Hospital, P.O. Box 16, Tamale, Ghana; ^3^School of Medicine, Wake Forest University, Winston-Salem, NC, USA

## Abstract

*Objective.* This study investigated the treatment outcomes and determinant factors likely to be associated with recovery rate. *Methods.* A retrospective chart review (RCR) was performed on 348 patients who were enrolled in the outpatient care (OPC) during the study period. *Results.* Of the 348 cases, 33.6% recovered (having MUAC ≥125 mm), 49.1% defaulted, and 11.5% transferred to other OPC units to continue with treatment. There were 187 (53.7%) males and 161 (46.3%) females with severe malnutrition. The average weight gain rate was 28 g/kg/day. Controlling for other factors, patients who completed the treatment plan had 3.2 times higher probability of recovery from severe acute malnutrition (SAM) as compared to patients who defaulted (adjusted odds ratio (AOR) = 3.2, 95% CI = 1.9, 5.3, and *p* < 0.001). The children aged 24–59 months had 5.8 times higher probability of recovery from SAM as compared to children aged 6–11 months (AOR = 5.8, 95% CI = 2.5, 10.6, and *p* < 0.001). *Conclusions.* Cure rate was low and the default rate was quite high. Children who were diagnosed as having marasmus on admission stayed longer before recovery than their kwashiorkor counterparts. Younger children were of greater risk of nonrecovery.

## 1. Introduction

Severe acute malnutrition (SAM) affects nearly 20 million children under five and contributes to one million child deaths yearly [1, 2]. SAM is an important cofactor in the development of severe infections. SAM as defined by WHO-UNICEF includes severe wasting and nutritional oedema. Severe wasting (marasmus) is defined as weight-for-height (WH) below −3 standard deviations (SD or *Z*-scores) or mid-upper arm circumference (MUAC) <115 mm [2–4].

A child with SAM has a limited ability to respond to stressors (infection and environmental), is highly vulnerable, and has a high mortality risk [5, 6]. Severely underweight and wasted children had an approximately eight- to nine-fold increased risk of mortality as shown in, respectively, [7] and [1]. Stunting, severe wasting, and intrauterine growth restriction together are responsible for 2.2 million deaths per year and 21% of disability-adjusted life-years for children younger than 5 years [1]. It is critical that such children are treated proactively with intensive treatment regimes of short duration, aiming to rehabilitate the child in a few weeks.

SAM is a common indication for hospital admission among pediatric patients in sub-Saharan Africa. In Ethiopia, severe acute malnutrition is the primary diagnosis in 20% of pediatric hospital admissions [8], while 41.4% of preschool-aged children are affected by malnutrition of any degree [9].

Relatively little has been published on treatment outcomes for SAM in outpatient care settings (OPC). One study in rural Malawi demonstrative of children enrolled in an outpatient treatment programme for moderate acute malnutrition demonstrated that 80% recovered, 4% defaulted, and 0.4% died [10]. In the same setting, 30% of children who completed treatment for moderate acute malnutrition either relapsed or died within one-year following treatment [11]. In northwest Ethiopia, patients hospitalized for severe malnutrition had a case fatality rate of 18%, and 9% abandoned treatment [12]. However, it is unknown how these results translate to in other regions of Africa.

UNICEF supports the rehabilitation of SAM cases in Ghana with Plumpy'Nut which is a ready-to-use therapeutic food (RUTF). Plumpy'Nut is energy, mineral, and vitamin enriched paste-food designed to treat SAM. A sachet has a serving size of 92 gm and gives energy of 2,100 kJ (500 kcal). Severely malnourished children are also provided with routine medications such as deworming tabs, antibiotics, vitamin A, folic acid, and measles vaccine. Only children who fulfil the criteria for SAM, who do not have medical complications, and who have passed the appetite test with Plumpy'Nut are managed at the OPC. Once admitted, children get a weekly Plumpy'Nut ration. They receive different amount of Plumpy'Nut sachets according to their body weight and appetite. They are also given routine medications during the course of the treatment such as vitamin A, folic acid tabs, antibiotics, deworming tabs, and measles vaccine.

On each visit, children receive medical review. This includes temperature, respiratory rates, and pulse measurements. Medical history is also taken every week and that includes history of vomiting, diarrhoea, cough, and temperature in the last 7 days. Nutrition education is also given to mothers or caretakers. They are counseled on how to provide adequate diets for their children at home in order to prevent reoccurrence of malnutrition or other siblings from becoming malnourished.

The treatment of SAM cases in outpatient care (OPC) has been going on since 2011 in the TTH; however little is known about the treatment outcomes and factors determining the recovery rate of the children presenting with SAM in the OPC. This study sought to assess the performance of the programme and determinants of recovery rate in an Outpatient Paediatric Nutrition Clinic in Northern Ghana.

## 2. Materials and Methods

### 2.1. Study Site

All study patients were enrolled from the OPC of the Tamale Teaching Hospital in Tamale, Ghana. This hospital has a 452-bed capacity and serves as a major referral center for Northern Ghana, with an estimated catchment population of 2.1 million.

### 2.2. Study Design

We performed a retrospective chart review (RCR) of patients enrolled in the outpatient care (OPC) clinic 2011–2013 at the Tamale Teaching Hospital.

### 2.3. Study Population Selection (Exclusion Criteria)

No sampling was done but all malnourished children aged 6–59 months who sought outpatient care between 2011 and 2013 were included in the study. However, those children with some variables not recorded were excluded from the study. A total of 353 outpatient cases were reviewed but 348 patients had a complete set of data and were included in the analysis.

### 2.4. Outcome Measures

The main outcome indicators were average length of stay, average rate of weight gain, cure rate, death rate, default rate, and transfer rate.

### 2.5. Data Collection

The source of data for the study was individual OPC record documents including registers and monitoring cards. Information extracted were patient age, sex, residence, admission criteria, the number of admissions, death, defaulters, date of admission and discharge, length of stay, diagnosis, and discharge condition (resolved malnutrition, death, and lost to follow-up). Additionally, anthropometric measurements, including weight, presence of bilateral oedema, and mid-upper arm circumference, were collected at the time of enrollment, at discharge, and during treatment.

The average length of stay was calculated by adding the total number of days that each child discharged as cured stayed in the OPC and dividing this by the number of children cured for a specific month.

The length of stay (LOS) in the OPC and the rate of weight gain were computed only for those children admitted with marasmus and who recovered from SAM. This is methodology within the Guidelines of the International SPHERE standard [13, 14].

Rate of weight gain was calculated using the formula

(1) $$\begin{array}{c} \frac{\text{Discharge Weight (g)}-\text{Minimum Weight (g)}}{\text{Minimum Weight}\times \text{no. of days between minimum weight and discharged weight}}. \end{array}$$

### 2.6. Data Processing and Analysis

Data was checked for correctness and consistency and were analyzed using SPSS for Windows (version 21; SPSS Inc., Chicago, IL, USA). First, frequency tables were produced for different variables and cross tables were produced accordingly. Mean values were produced for continuous variables. Comparison between groups was done using chi-square tests for proportions and *t*-tests or ANOVA procedures for continuous variables. Regression analysis was performed to identify independent outcome predictors.

A trend analysis was carried out to help assess if severe malnutrition cases are increasing or declining over time. Seasonal changes in severe malnutrition cases were also investigated.

### 2.7. Ethics Consideration

Ethical approval was obtained from the Tamale Teaching Hospital with reference number TTH/R&M/SR/13/91. In this study there was no direct contact with patients and secondary data was used anonymously by using identity numbers instead of names in order to protect patient identity. As this was a retrospective chart review, there was minimal risk involved to participants. All protected health information (PHI) was deidentified prior to data analysis and publication; subject identities were known only to the study staff. No reference to any individual participant is made in the study reports.

To help ensure subject privacy and confidentiality, only a unique study identifier appeared on the data collection form for each subject. Any collected subject identifying information corresponding to the unique study identifier was maintained on a linkage file, stored separately from the data. Data access was limited to study staff.

## 3. Results

### 3.1. Sample Characteristics

The mean ± SD age of the children was 19.2 ± 10.9 months, with 36.8% being in the age groups of 12–23 and 24–59 months. There were 187 (53.7%) males and 161 (46.3%) females with severe malnutrition. Children below the age of 24 months accounted for 63.2% of all the admissions. Overall, 67.2% of cases met the criteria for SAM (MUAC <115 mm), 27.3% of cases met the criterion for moderate acute malnutrition (115 to <125), and 5.5% were normal (MUAC ≥125 mm). This latter category of children with normal MUAC could have had some form of oedema suggesting SAM cases. The mean MUAC of children on admission and discharge was 11.1 ± 0.9 and 11.9 ± 1.2, respectively. The average number of days spent in the facility was 7.0 ± 5.4 with the minimum and maximum being 1 and 47 days, respectively. Well over 67% of cases admitted to the OPC were referred from the inpatient care (IPC) of the same hospital (Table 1).

Based on MUAC, 25.5% SAM cases on admission progressed to a normal nutritional status whereas 24.8% improved to moderate acute malnutrition. Similarly, the proportion of moderate acute malnutrition that converted to normal status was 40.0% on discharge (Table 2).

### 3.2. Trend in Admissions in the OPC

**Figure 1:**
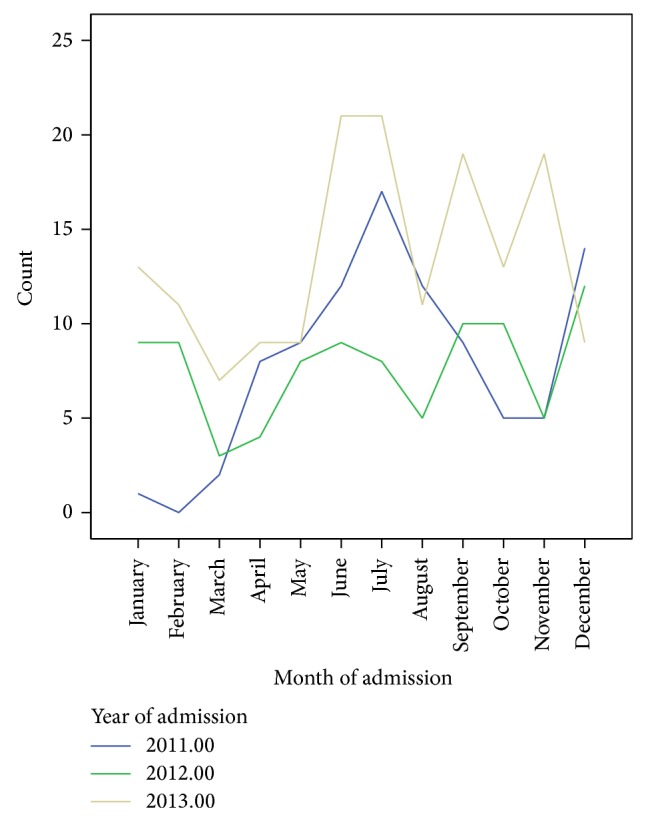
Trends in admissions in the OPC.

The trend analysis was carried out to help assess if severe malnutrition cases are increasing or declining over time. Seasonal changes in severe malnutrition cases were also investigated as shown in Figure 1. The peak months for admissions over the three-year period were June and July and the highest number of admissions was recorded in 2013. The least number of cases was admitted in March.

There was no significant difference in the trend of admissions of malnutrition cases in terms of sex over the three-year period (Table 3).

### 3.3. Programme Effectiveness as Gauged by the Global SPHERE Standards

The main outcome indicators were cure rate, death rate, default rate, and transfer rate. The treatment outcomes of severely malnourished children from 2011 to 2013 at the OPC are shown in Table 4. In all, 348 children aged 6–59 months with SAM were admitted to the OPC during the period of study. Of these, 33.6% recovered (having MUAC ≥125), 49.1% defaulted, and 11.5% transferred to other OPC units to continue with treatment. The case fatality rate was 0.0%.

Among SAM cases who were cured, the average length of stay was 8.0 ± 5.34 days and the maximum was 33 days (4 weeks). The children who recovered from SAM had an average weight gain of 28.3 ± 23.9 gm/kg/day.

Children that were diagnosed with kwashiorkor (presence of bilateral pitting oedema) on admission seemed to have greater weight and MUAC on discharge than their marasmic counterparts but the average length of stay was not significantly different among children who were discharged as cured (Table 5).

### 3.4. Factors Associated with the Type of Malnutrition on Admission

Marasmic children came more from an urban setting whilst the kwashiorkor came more from rural settings. Marasmus was more predominant in dry season compared to the rainy season (96.4% versus 86.1) (Chi = 8.5, *p* = 0.004). Kwashiorkor was more prevalent in the rainy season than the dry season at the OPC (Table 6). However, no significant difference was observed in the occurrence of type of malnutrition between males and females.

### 3.5. Factors Associated with Recovery from Severe Acute Malnutrition

Bivariate and logistic regression analysis was performed to identify factors that independently predict recovery from SAM. In the bivariate analysis, the factors associated with recovery from SAM are shown in Table 7. No significant difference in recovery rate was observed with respect to where child was admitted from, place of residence, sex of the child, and duration of stay in the programme. Children aged 24–59 months were more likely to recover compared to children 6–11 months (Chi = 23.4, *p* < 0.001).

Controlling for other factors, the patients who did not default during treatment had 3.2 times higher probability of recovery from SAM as compared to the patients who defaulted (AOR = 3.2, 95% CI = 1.9, 5.3, and *p* < 0.001). Likewise, the children aged 24–59 months had 5.8 times higher probability of recovery from SAM as compared to children aged 6–11 months (AOR = 5.8, 95% CI = 2.5, 10.6, and *p* < 0.001) (Table 8).

The set of factors accounted for 23.1% of the variance in cure or recovery rate (Nagelkerke *R* square = 0.231).

## 4. Discussion

A MUAC of less than 115 mm and/or bipedal oedema are currently the only admission criteria for the programme. This study analyzed the treatment outcomes of infants and children aged 6–59 months and who have a mid-upper arm circumference <115 mm and/or the presence of bilateral oedema (swelling of both feet). These children were admitted for the management of severe acute malnutrition.

Most of the children admitted were marasmic (MUAC <115 mm but without oedema). This is in agreement with what other studies have found that marasmus is more prevalent than kwashiorkor in Northern Ghana [15].

The aetiology of severe wasting (marasmus) is linked to the situation where the child consumes much less food than required for his or her energy needs and so energy is mobilized from both body fat and muscle. Gluconeogenesis in the liver is enhanced, and there is loss of subcutaneous fat and wasting of muscles.

### 4.1. Treatment Outcomes

The effectiveness of an OPC programme is gauged by the Global SPHERE standards. The defaulter and recovery rates in this study were outside the acceptable range of Global SPHERE standards [13]. However, the average rate of weight gain was substantially more than the International SPHERE standard.

The cure rate was quite low whereas the defaulter rate was three times higher than the acceptable SPHERE standard. A defaulter is a patient that is absent for two consecutive weeks and it is confirmed that the patient is not dead. It is unclear what factors contribute to the very high defaulter rate observed in this study (49.1%). The less than 40% recovery rate may be directly attributable to the 50% defaulter rate and the significant number of cases transferred to other outpatients' clinics. The outcomes for these patients are unknown and limit a complete interpretation of the data for this programme. No significant age and sex difference was found in terms of default rate.

If malnourished children access nutritional care early in the onset of their condition and comply with treatment until they have recovered, one might expect improved medical and nutritional outcomes. Conversely, if patients access care late and/or they are deterred from staying in the programme for as long as necessary, then success rates will be low. To achieve the Millenium Development Goal 4, reduction in child mortality [16], the management of SAM needs to be implemented correctly. This means that outpatient programmes should decrease barriers to access, encourage early identification of malnutrition, reduce inpatient caseloads and so decrease the risks of cross infection, reduce costs associated with treatment, encourage compliance by patients, and increase the time available to staff to help the sickest children [17, 18].

The rate of weight gain observed among marasmic children who recovered was 28.3 gm/kg/day. This is relatively high when compared to the goals set by the SPHERE standards (Table 4). Usually, a weight gain of 10–15 g/kg/day is considered satisfactory; and conversely if the weight gain is less than 5 g/kg for three consecutive days, it shows that the child is not responding to the treatment. The discharge weight is usually seen as 90% of expected weight for age and most children reach this target weight within two to four weeks of therapy [19].

### 4.2. Patient Factors That Might Have Contributed to Patient Outcome

Among SAM cases who were cured, the average length of stay was 8.0 ± 5.34 days and the maximum was 33 days (4 weeks). SAM cases who were admitted on the basis of having oedema (i.e., kwashiorkor) recovered faster than cases that were admitted based on low MUAC. Those children who were diagnosed with marasmus on admission stayed longer (LOS) before recovery than their kwashiorkor counterparts (LOS). This finding is consistent with the findings of earlier studies [20, 21].

Children with severe acute malnutrition should only be discharged from treatment when their weight-for-height/length is ≥−2 *Z*-score and they have had no oedema for at least 2 weeks, or mid-upper arm circumference is ≥125 mm and they have had no oedema for at least 2 weeks [22].

## 5. Conclusion

This study demonstrates a high defaulter rate but a recovery rate of 34% from severe malnutrition in our Outpatient Clinic. The mean weight gain was 28 gm/kg/day with a mean LOS of 8 days. These results are in concordance with the goals of the SPHERE standards and WHO criteria for discharge from nutritional programmes. The study was limited by a high default rate of 49% of patients. Reasons for default from the programme were not identified. The findings reinforce the need to curb default during management of severe malnutrition. It is thus recommended that a study be done which may include interviews with key informants such as programme managers and focus group discussions with caretakers to come up with strategies that may help to reduce the default rate and improve on recovery rate. Overall mortality rate of those completing the programme is 0.0%.

## Figures and Tables

**Table 1:** Sociodemographic characteristics of study sample (*N* = 348).

Characteristics	*N*	%
Age (months)		
6–11	92	26.4
12–23	128	36.8
24–59	128	36.8
Total	**348**	**100.0**
MUAC (mm) on admission		
Severe (<115)	234	67.2
Moderate (115 to <125)	95	27.3
Normal (≥125)	19	5.5
Total	**348**	**100.0**
MUAC (mm) on discharge		
Severe (<115)	119	34.2
Moderate (115 to <125)	112	32.2
Normal (≥125)	117	33.6
Total	**348**	**100.0**
Duration in OPC (days)		
1–14	323	92.8
**15**–**28**	21	6.0
29–42	3	0.9
>42 days	1	0.3
Total	**348**	**100.0**
Admission		
Direct from community	79	22.7
Referred from health centre	34	9.8
Referred from inpatient care	235	67.5
Total	**348**	**100.0**

**Table 2:** Cross-tabulation between mid-upper arm circumference (MUAC) on admission and MUAC at discharge.

		MUAC at discharge			Total
		Severe	Moderate	Normal	
MUAC on admission					
Severe (<11.5 cm)	Count	116	58	60	234
	%	49.6	24.8	25.6	100.0
Moderate (11.5 to <12.5 cm)	Count	3	54	38	95
	%	3.2	56.8	40.0	100.0
Normal (at least 12.5 cm)	Count	0	0	19	19
	%	0.0	0.0	100.0	100.0
Total	Count	**119**	**112**	**117**	**348**
	%	**34.2**	**32.2**	**33.6**	**100.0**

**Table 3:** Annual and seasonal trends in admissions in the OPC (bivariate analysis).

Factor	*N*	Sex of child		Test statistic
		Male *n* (%)	Female *n* (%)	
Year of admission				
2011	94	48 (51.1)	46 (48.9)	
2012	92	52 (56.5)	40 (43.5)	Chi-square (*χ* ^2^) = 0.6, *p* = 0.8
2013	162	87 (53.7)	75 (46.3)	
Season of admission				
Dry	111	60 (54.1)	51 (45.9)	*χ* ^2^ = 0.0, *p* = 0.94
Rainy	237	127 (53.6)	110 (46.4)	

**Table 4:** Comparison of the treatment outcomes of severely malnourished children at the outpatient care (OPC) with international SPHERE standards (*N* = 348).

Outcome indicator	Outcome (%)	SPHERE standards	
		Acceptable	Alarming
Recovery rate	33.6	>75%	<50%
Nonresponse rate	0.3	15	
Defaulter rate	49.1	<15%	>25%
Case fatality rate (CFR)	0.0	<10%	>15%
Average rate of weight gain (g/kg/day)	28.2	≥8	<8
Average length of stay (weeks)	1.0	<8 weeks	>6 weeks
Referred to IPC	0.3	—	—
Transferred to other OPC	11.5	—	—

**Table 5:** Length of stay and treatment outcomes among cured children under different admission criteria.

Outcome/admission criteria	*N*	Mean	Std. deviation	95% confidence interval for mean		Test statistic
				Lower bound	Upper bound	
MUAC on discharge						
MUAC < 115 mm	98	13.2	0.66	13.04	13.31	*F*(1,116) = 4.6, *p* = 0.04
Bilateral pitting oedema	19	13.6	1.15	13.03	14.14	
Total	**117**	**13.2**	**0.77**	**13.10**	**13.38**	
Discharge weight						
MUAC < 115 mm	98	8.8	1.83	8.47	9.21	*F*(1,116) = 6.2, *p* = 0.01
Bilateral pitting oedema	19	9.9	1.31	9.30	10.57	
Total	**117**	**9.0**	**1.80**	**8.69**	**9.35**	
Number of days on treatment						
MUAC < 115 mm	98	8.4	5.13	7.35	9.41	*F*(1,116) = 0.005, *p* = 0.9
Bilateral pitting oedema	19	8.5	5.91	5.63	11.32	
Total	**117**	**8.4**	**5.24**	**7.43**	**9.35**	

**Table 6:** Factors associated with the type of malnutrition on admission.

Factor	*N*	Type of malnutrition		Test statistic
		Marasmus *n* (%)	Kwashiorkor *n* (%)	
Age (months)				
6–11	92	90 (97.8)	2 (2.2)	Chi-square (*χ* ^2^) = 24.3, *p* < 0.001
12–23	128	120 (93.8)	8 (6.3)	
24–59	128	101 (78.9)	27 (21.1)	
Total	**348**	**311 (89.4)**	**37 (10.6)**	
Season of admission				
Dry	111	107 (96.4)	4 (3.6)	*χ* ^2^ = 8.5, *p* = 0.004
Rainy	237	204 (86.1)	33 (13.9)	
Total	**348**	**311 (89.4)**	**37 (10.6)**	
Place of residence				
Urban	201	187 (93.0)	14 (7.0)	*χ* ^2^ = 6.7, *p* = 0.009
Rural	147	124 (84.4)	23 (15.6)	
Total	**348**	**311 (89.4)**	**37 (10.6)**	
Gender				
Male	187	169 (90.4)	18 (9.6)	*χ* ^2^ = 0.4, *p* = 0.5
Female	161	142 (88.2)	19 (11.8)	
Total	**348**	**311 (89.4)**	**37 (10.6)**	

**Table 7:** Factors associated with recovery from severe acute malnutrition (bivariate analysis).

Factor	*N*	Recovery from SAM?		Test statistic
		No *n* (%)	Yes *n* (%)	
Age group (months)				
6–11	92	77 (83.7)	15 (16.3)	
12–23	128	81 (63.3)	47 (36.7)	Chi-square (*χ* ^2^) = 17.9, *p* < 0.001
24–59	128	73 (57.0)	55 (43.0)	
Admission criteria				
MUAC < 115 mm	311	213 (68.5)	98 (31.5)	*χ* ^2^ = 5.8, *p* = 0.02
Bilateral pitting oedema	37	18 (48.6)	19 (51.4)	
Classification of % change in weight				
Up to 20%	256	187 (73.0)	69 (27.0)	*χ* ^2^ = 19.2, *p* < 0.001
More than 20%	92	44 (47.8)	48 (52.2)	
Defaulted				
No	163	83 (50.9)	80 (49.1)	*χ* ^2^ = 32.8, *p* < 0.001
Yes	185	148 (80.0)	37 (20.0)	

**Table 8:** Factors associated with recovery from severe acute malnutrition (multivariate analysis).

	Wald	Sig.	Exp(*β*)	95% CI for EXP(*β*)	
				Lower	Upper
Not defaulted during treatment	18.99	<0.001	3.2	1.9	5.3
>20% change in weight	10.84	0.001	2.6	1.5	4.6
Age group (reference: 6–11 months)	20.12	<0.001			
12–23 months	10.35	0.001	3.2	1.6	6.5
24–59 months	20.12	<0.001	5.8	2.5	10.6
Constant	54.91	<0.001	0.07		
